# Thermochromic Gires‐Tournois Resonators with Tellurium for Battery Thermal Runaway Warning

**DOI:** 10.1002/adma.202511261

**Published:** 2025-07-23

**Authors:** Hyun Min Kim, JuHyeong Lee, Juhwan Kim, Gyurin Kim, Jang‐Hwan Han, Joo Hwan Ko, Young Min Song, Hyeon‐Ho Jeong

**Affiliations:** ^1^ Department of Electrical Engineering and Computer Science Gwangju Institute of Science and Technology Gwangju 61005 Republic of Korea; ^2^ Department of Semiconductor Engineering Gwangju Institute of Science and Technology Gwangju 61005 Republic of Korea; ^3^ Artificial Intelligence (AI) Graduate School Gwangju Institute of Science and Technology Gwangju 61005 Republic of Korea; ^4^ School of Electrical Engineering Korea Advanced Institute of Science and Technology (KAIST) Daejeon 34141 Republic of Korea

**Keywords:** battery thermal runaway warning, Gires‐Tournois resonator, tellurium, thermochromic materials

## Abstract

Effective temperature monitoring is crucial for preventing battery fires caused by thermal runaway, ensuring human safety, and providing timely warnings. While thermochromic materials offer intuitive, real‐time temperature visualization, their slow response times remain them unsuitable for battery monitoring. A thermochromic Gires‐Tournois (GT) resonator specifically designed for rapid and accurate battery temperature detection in the critical range below 80 °C is introduced, where thermal runaway risks can be effectively mitigated. Central to this design is an ultrathin (10 nm) thermo‐responsive tellurium film, paired with a protective glass layer and an underlying metallic mirror. This thermochromic GT resonator exhibits reversible temperature detection over multiple cycles, actively responding to temperature changes through partial melting of tellurium, which alters its complex refractive index—a property discovered in the 1960s but now harnessed for this novel application. Notably, the resonator monitors both specific temperature points and overall heat transfer across the battery surface, achieving sub‐second response times in an untethered manner. These findings position the thermochromic GT resonator as a promising platform for direct, intuitive, and compact temperature monitoring in energy storage systems.

## Graphical abstract

A thermochromic Gires‐Tournois (GT) resonator with a thin tellurium layer enables rapid, reversible, and untethered detection of battery surface temperature <80 °C. Exploiting tellurium's refractive index change driven by a thermally induced phase transition between solid and quasi‐liquid states, the GT resonator visualizes heat propagation, providing an effective early warning system against battery thermal runaway.

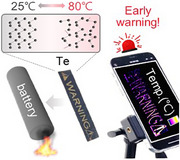

## 1 Introduction

The rapid transition from the electric to artificial intelligence (AI) era has accelerated the development of various AI‐assisted electric personal items (such as mobile phones, clocks, rings, etc.) and vehicles (e.g., electric cars and urban air mobility), all of which essentially require portable energy storage and conversion devices, commonly known as batteries.^[^ ^1^, ^2^ ^]^ Despite their commercial success, batteries are prone to recurrent fire accidents, which pose severe risks to both personal and public property, as well as to human life, and thus cause significant social concern and conflict between users and non‐users alike.^[^ ^3^ ^]^ Specifically, an uncontrollable temperature increases within a battery cell, termed thermal runaway, can occur due to various factors (such as mechanical damages, electrical overload, and thermal abuse), leading to a chain reaction of exothermic events.^[^ ^4^ ^]^ This reaction can exponentially elevate the temperature to over 500 °C in less than a minute, often resulting in unpredictable fires and explosions.^[^ ^5^ ^]^

**Figure 1:**
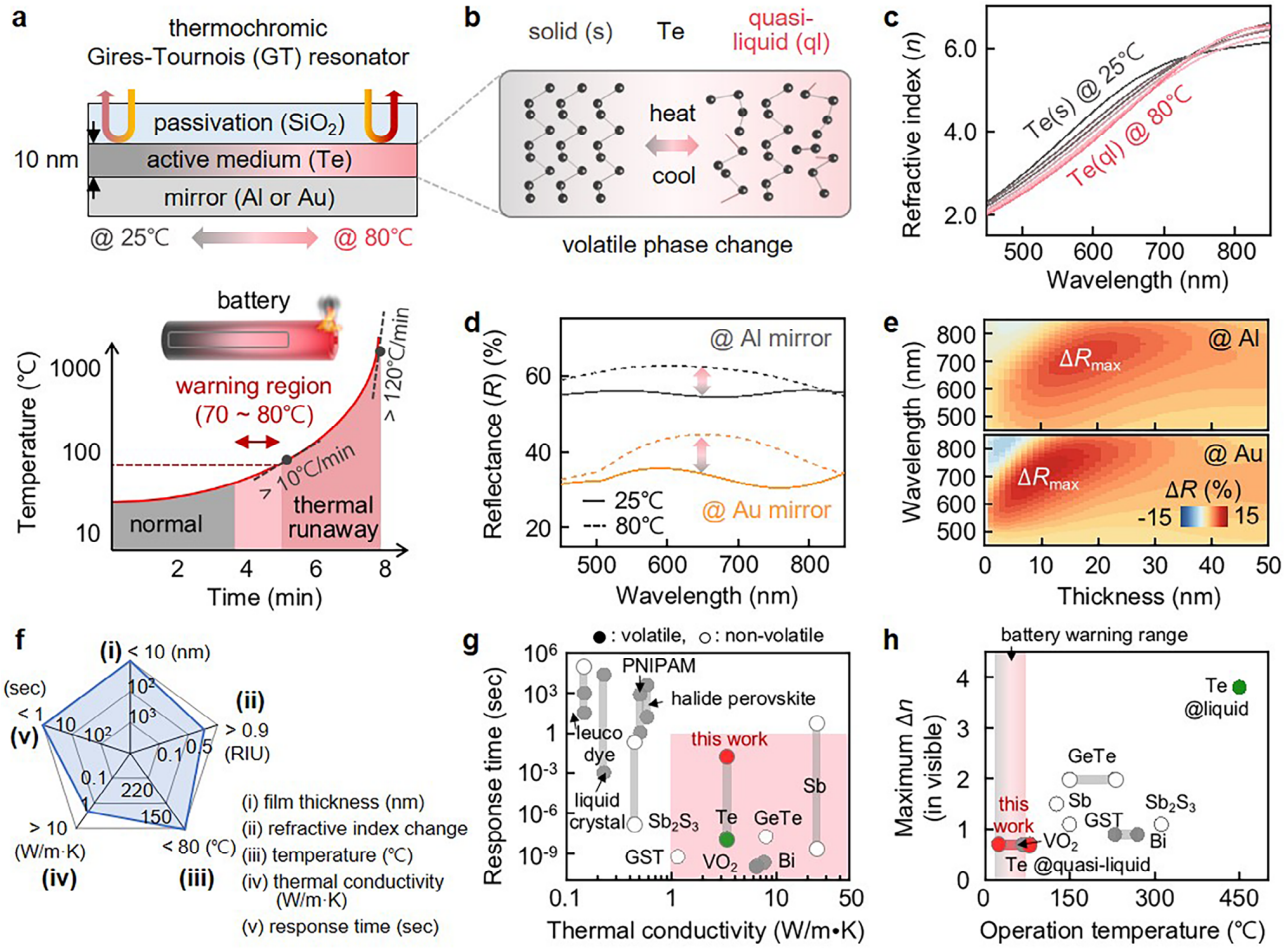
Thermochromic Gires‐Tournois (GT) resonators with elemental tellurium (Te). a) Schematic of the thermochromic GT resonator integrated onto a battery surface (top) to visually indicate temperature changes linked to the battery's operational status (bottom). b) Thermo‐responsive phase transition of Te between solid and quasi‐liquid states. c) Corresponding changes in the refractive index during the phase transition. d) Numerically calculated reflection spectra of the GT resonators (gray: Al mirror, yellow: Au mirror) as the temperature increases from 25 to 80 °C. e) Associated calculated reflection difference (Δ*R*) versus Te film thickness. f) Performances of the thermochromic GT resonator and g) its comparison with 11 different thermochromic materials for the response time versus thermal conductivity, and h) maximum change in refractive index versus operation temperature (dot: volatile, circle: non‐volatile).^[^ ^15^, ^16^, ^17^, ^18^, ^19^, ^20^, ^21^, ^22^, ^23^, ^24^, ^25^, ^26^, ^27^, ^28^, ^29^, ^30^, ^31^, ^32^, ^33^, ^34^, ^35^, ^36^, ^37^, ^38^, ^39^, ^40^, ^41^, ^42^, ^43^, ^44^ ^]^

A rational approach to prevent such dangerous temperature spikes and associated hazards is to detect early signs of overheating before thermal runaway, typically when the battery temperature is below 80 °C (e.g., **Figure** **1a**).^[^ ^4^, ^5^, ^6^ ^]^ Once the battery temperature exceeds 80 °C due to mechanical impact, short circuit, overcharging, or external heat, critical damage can occur to internal components, such as the solid electrolyte interface and the separator, triggering rapid thermal runaway. So, for early detection, thermocouples and infrared (IR) camera are widely used due to their fast response times (below 1 sec).^[^ ^7^, ^8^, ^9^ ^]^ However, thermocouples need to be physically attached to the exterior of the battery, thus only measuring temperature at the specific local area of contact.^[^ ^10^ ^]^ Additionally, in large‐scale battery packs containing hundreds of cells, the number of thermocouples is typically limited due to cost and integration complexity.^[^ ^11^ ^]^ Conversely, IR cameras provide spatially resolved thermal maps in an untethered configuration but are often hindered by inaccuracies arising from variations in surface emissivity and background interference.^[^ ^12^ ^]^

As an alternative, thermochromic materials offer intuitive and contactless visualization of temperature distributions with minimal instrumentation (see Supplementary Table S1, Supporting Information).^[^ ^13^, ^14^ ^]^ For instance, organic molecules, such as leuco dyes and liquid crystals, can exhibit significant color changes with varying temperatures.^[^ ^15^, ^16^, ^17^ ^]^ However, their response times exceed 1 min,^[^ ^18^, ^19^, ^20^, ^21^ ^]^ making them unsuitable for preventing rapid thermal events. Similarly, halide perovskite materials offer large color changes from red to green as the temperature changes from 20 to 60 °C, but yet suffer from slow response times (above 30 min) and poor thermal stability.^[^ ^22^, ^23^ ^]^ In contrast, inorganic phase‐change materials are known for their extremely fast response times, often within tens of nanoseconds,^[^ ^24^, ^25^, ^26^, ^27^, ^28^, ^29^, ^30^ ^]^ and exhibit a large thermo‐responsive change in refractive index (*Δn*) exceeding 0.5.^[^ ^31^, ^32^, ^33^ ^]^ Antimony (Sb), for example, has emerged as a promising thermochromic material with high thermal conductance (≈4860 MW m^−^^2^∙K^−1^ for a 5 nm film) and large optical tunability (*Δn* > 1.5).^[^ ^33^ ^]^ However, Sb‐based systems rely on irreversible crystalline phase transitions, which typically occur above 80 °C, making them incompatible with early‐stage battery thermal monitoring.^[^ ^33^, ^34^, ^35^, ^36^ ^]^ Moreover, these transitions are nonvolatile,^[^ ^37^, ^38^ ^]^ and susceptible to structural variability due to oxygen vacancies and stoichiometry‐dependent polymorphism,^[^ ^24^, ^39^ ^]^ limiting their reproducibility and cycling stability.

We here propose tellurium (Te) as a novel single‐element volatile phase‐change material, effective for thermochromic devices in battery applications. Unlike other inorganic phase‐change materials, the atomic structure of Te sequentially melts from solid to quasi‐liquid phase as the temperature increases to 80 °C, effectively tuning its refractive index over 0.7 in the visible range. We leverage this property to develop an ultrathin Gires‐Tournois (GT) resonator, requiring only 10 nm thick Te film on a metallic surface (such as aluminum, commonly used for the battery casings). This resonator effectively tunes the reflectance by 2% with small temperature variations within seconds. We further demonstrate that these thermochromic GT resonators enable rapid and reversible switching, making them suitable for integration on the surface of the battery cells and thus providing real‐time visualization of thermal fluctuations in space, a critical feature for early thermal runaway warnings.

## 2 Results and Discussion

### 2.1 Design of Gires‐Tournois (GT) Resonators with Tellurium (Te)

To rapidly and reversibly detect the battery temperature in the critical “warning region” below 80 °C, while providing an intuitive visual indication (Figure 1a), thermochromic materials must meet two essential criteria (Figure S1, Supporting Information): (i) a fast response time under 1 min, enabled by high thermal conductivity, a criterion that most organic phase‐change materials fail to meet,^[^ ^13^, ^40^ ^]^ and (ii) a large, reversible change in refractive index in response to external thermal input within the visible range, which is inadequately provided by most inorganic phase‐change materials.^[^ ^31^, ^37^ ^]^ To address these challenges, we introduce an elemental Te as a thermochromic material that satisfies the stringent demands of battery temperature monitoring. When transitioning between solid and liquid phases (Figure 1b), Te exhibits an exceptionally large thermo‐responsive shift in its refractive index, exceeding 3 in the visible spectrum.^[^ ^41^, ^42^ ^]^ This significant optical property, first reported in the 1960s, has been surprisingly overlooked despite being the highest known refractive index transition in the visible range.^[^ ^42^ ^]^ Additionally, Te intrinsically possesses superior thermal conductivity (*c.a*. 3.38 W m^−1^·K^−1^),^[^ ^43^ ^]^ resulting in an extremely fast phase transition time of 12 nanoseconds.^[^ ^44^ ^]^ Furthermore, it has been recently explored as an electrode material in lithium‐ion batteries,^[^ ^45^, ^46^ ^]^ indicating its feasibility and compatibility for integration into battery temperature sensing applications.

Leveraging such Te's thermo‐responsive properties, we develop a simple yet highly efficient thermochromic ultrathin reflector, referred to as a GT resonator.^[^ ^47^ ^]^ Prior to the structural design, we validate the changes in Te's refractive index within the warning region near 80 °C using *in*‐*situ* ellipsometry while heating the sample (Figure 1c). As the temperature rises from 25 to 80 °C, the refractive index (*n*) of the Te thin film decreases across the visible spectrum at wavelength λ < 750 nm, while the extinction coefficient (*k*) increases (Figure S2, Supporting Information). Using these optical properties, we then design and simulate tailorable GT resonators for the temperature visualization using a tri‐layer configuration of glass/Te/metallic mirror (top panel of Figure 1a). Since Te behaves optically as a lossy dielectric, similar to silicon and germanium,^[^ ^48^ ^]^ the combination of a thin Te layer with an underlying metallic (Au or Al) mirror enables strong optical interference, despite the subwavelength thickness.^[^ ^49^ ^]^ Due to its exceptional thermal and chemical stability,^[^ ^50^ ^]^ the Au mirror is primarily used to eliminate mirror‐induced changes and focus solely on the thermochromic behavior of the Te film, while the Al mirror is utilized for battery applications. For example, at *T* = 25 °C, a 10 nm thick solid Te film formed on an Au mirror reflects 34% of light at λ = 650 nm, which increases to over 40% as Te partially liquifies at 80 °C (Figure 1d). This thermochromic behavior remains consistent regardless of mirror material, as shown for both Au and Al mirrors. Moreover, the reflectance difference (*ΔR*) between solid and partial liquid (*a.k.a*. quasi‐liquid) phases is prominent across all Te film thicknesses ranging from 1 to 50 nm (Figure 1e). We here define the *ΔR* as the difference between the reflectance of the GT resonators at a given temperature and 25 °C. Notably, simulations suggest that the GT resonators made of 10 nm Te film exhibit the maximal *ΔR* in visible and exceptional thermal conductance (≈338 MW m^−2^·K^−1^), one of the highest values recorded, ensuring rapid thermal response (Figures 1f–h; Figure S1 and Table S1, Supporting Information). Additionally, the upper silica layer, which fully covers the entire surface of the GT resonator, serve a dual purpose (see below): it not only prevents Te evaporation without inducing alloying or other chemical reactions during heating,^[^ ^51^ ^]^ but it also functions as a heat insulator that accelerates Te melting, thanks to its low thermal conductivity of ≈1 W m^−1^·K^−1^.^[^ ^40^, ^52^ ^]^

### 2.2 Operation of Thermochromic GT Resonators

**Figure 2:**
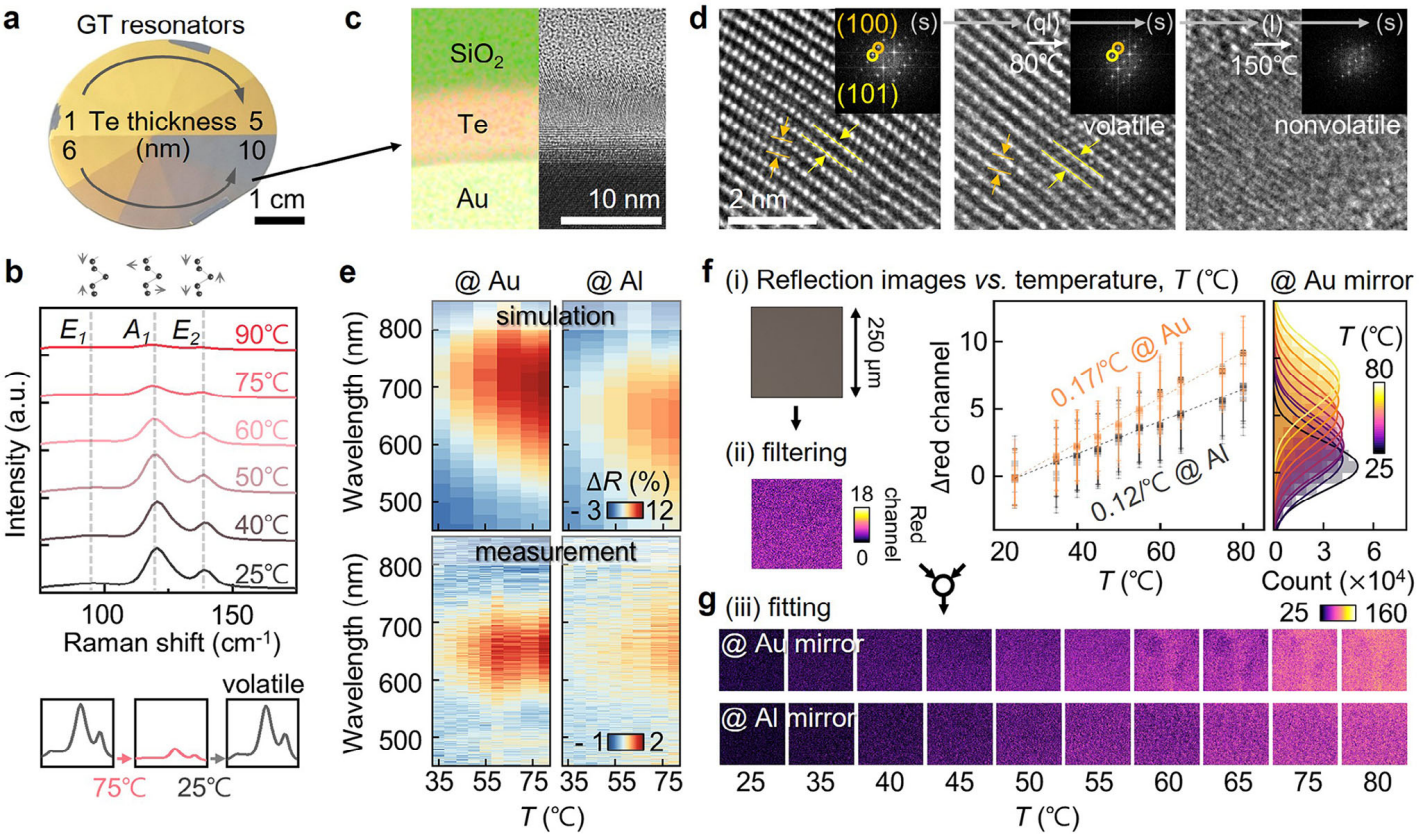
Operation of GT resonators below the Te melting point. a) Wafer‐scale GT resonators with different Te layer thicknesses. b) *In*‐*situ* Raman scattering spectra of the GT resonator during heating. c) Transmission electron microscopy (TEM) images of the GT resonator (right) and corresponding EDX elemental false color mapping (O: green, Te: red, Au: yellow). d) Transition of Te crystal structure with increasing temperature (inset: diffraction pattern). e) Reflection difference (*ΔR*) of the GT resonator fabricated on an Au mirror (top: simulation, bottom: measurement). f) Changes in reflection images of the GT resonator with temperature, including analysis of the separated red channel images. g) Temperature visualization using the GT resonator.

Based on numerical simulations, we fabricate GT resonators by depositing a 10 nm thick Te film onto an Au (or Al) mirror, protected by a 50 nm thick SiO_2_ layer, all of which are fabricated through physical evaporation techniques (see **Figure** **2a** and Experimental Section for the fabrication details). The key mechanism behind the GT resonator's thermal sensing capability lies in the phenomenon of partial melting, despite Te's bulk melting point being 450 °C.^[^ ^53^, ^54^ ^]^ We observe that melting begins at significantly lower temperatures, attributable to localized melting at the material's surface or grain boundaries prior to the complete phase transition.^[^ ^55^, ^56^, ^57^ ^]^ This enables the formation of a quasi‐liquid phase at temperatures well below the bulk melting point, allowing for temperature sensing based on variations in the liquid volume fraction. We confirm this process using Raman scattering analysis (Figure 2b) and transmission electron microscopy (TEM, Figure 2c,d). As the temperature increases from 25 to 90 °C, the Raman bands associated with intra‐chain bond bending (*E_1_*), chain expansion (*A_1_*), and bond stretching (*E_2_*) of Te chains progressively weaken (top panel of Figure 2b), indicating that the Te film is partially transitioning to the liquid phase.^[^ ^58^ ^]^ Notably, the Te film exhibits reversible transitions after repeated heating cycles at 75 °C, with negligible changes observed at room temperature, confirming its volatile nature (bottom panel of Figure 2b). TEM images and associated diffraction patterns further demonstrate that the initial trigonal crystal structure of the Te film remains intact at temperatures below 80 °C, suggesting that only specific regions of the Te film undergo melting, which automatically reverses upon cooling (Figure 2d). However, at temperatures above 150 °C, clear changes in the crystal structure occur, indicating irreversible melting, thus limiting the operational temperature range but still making the resonator well‐suited for battery warning applications. We further confirm the absence of structural deformation or alloy formation, such as Au‐Te or Si‐Te, in the GT resonator during heating, as evidenced by energy‐dispersive X‐ray spectroscopic (EDX) images (Figures S3,S4, Supporting Information). To further validate the thermally induced phase transition of Te, we measure the electrical resistance of Te thin film deposited on electrically insulating quartz substrates. As Te is known to exhibit increased electrical conductivity during its transition from solid to liquid,^[^ ^44^ ^]^ we observe a corresponding decrease in resistance with rising temperature, providing additional evidence of the phase transition (Figure S5, Supporting Information).

We then perform *in*‐*situ* reflection measurements of the GT resonators during heating, typically from 25 to 80 °C, using a customized optical spectroscopic system and imaging setup combined with a commercial microheater. At *T* = 35 °C, the GT resonators with Au mirror exhibit a reflectance change (Δ*R*) of 0.6% at λ = 650 nm (or 0.4% for the Al mirror), which increases by ≈2% as the temperature rises to 80 °C (Figure 2e; Figures S6,S7, Supporting Information). The overall trend of *∆R* variation aligns well with numerical simulation results based on the temperature‐dependent complex refractive indices of solid and quasi‐liquid Te, as measured by *in*‐*situ* heating ellipsometry. Discrepancies in the absolute *∆R* values are attributed to factors such as surface roughness, grain boundaries, and optical scattering in the fabricated films, which are not accounted for in the idealized simulation model. Nevertheless, the consistent trend supports the validity and predictive capability of the simulation. These dynamic optical changes are also visually evident. The red channel of the reflection images clearly shows intensity increases that correspond to the *∆R* changes (Figure 2f). To more effectively visualize spatial temperature fluctuations, we separate the primary RGB color channels from each reflection image and focus on the red channel, which exhibits the strongest temperature‐dependent variation in reflectance. The difference in red channel intensity (*Δred*) is then calculated by subtracting the red channel intensity at 25 °C from that at a given temperature and mapped pixel‐wise to construct thermal distribution profiles. This image‐based method enables clear and continuous visualization of temperature changes across the 25 to 80 °C range, as demonstrated in Figure 2g. Additionally, the GT resonators exhibit negligible color change at sub‐room temperatures (5, 10, and 15 °C) (Figure S8, Supporting Information), consistent with the absence of surface melting in this range. The temperature‐dependent thermochromic behavior of the GT resonators also closely follows the theoretically predicted trend of the liquid volume fraction of Te, which varies linearly between the solidus (25 °C) and liquidus (450 °C) states.^[^ ^59^ ^]^ This agreement supports the phase‐transition‐driven mechanism of the optical response and enables linear fitting of the color change with temperature (Figure S9, Supporting Information). Note that these thermochromic behaviors are solely due to the phase transition behavior of Te. Control experiments confirm that no observable color change occurs when using either an Au mirror alone or an Au mirror coated with a 50 nm SiO_2_ layer under identical heating conditions (Figure S10, Supporting Information). Furthermore, in the absence of the upper SiO_2_ layer, the Te film predominantly undergoes oxidation, forming TeO_x_ bonds, as confirmed by X‐ray photoelectron spectroscopy (Figure S11a, Supporting Information). In contrast, the Te film remains stable even after six months when protected by the SiO_2_ layer, which effectively acts as a passivation layer (Figure S11b, Supporting Information). This SiO_2_ layer also functions as a heat insulator, aiding the thermochromic response by trapping radiant heat and facilitating the partial melting of Te.^[^ ^52^ ^]^ This is further evidenced by the fact that unprotected GT resonators exhibit significantly smaller reflection changes compared to those with SiO_2_ protection (Figure S12, Supporting Information).

### 2.3 Temperature Visualization Using GT Resonators

**Figure 3:**
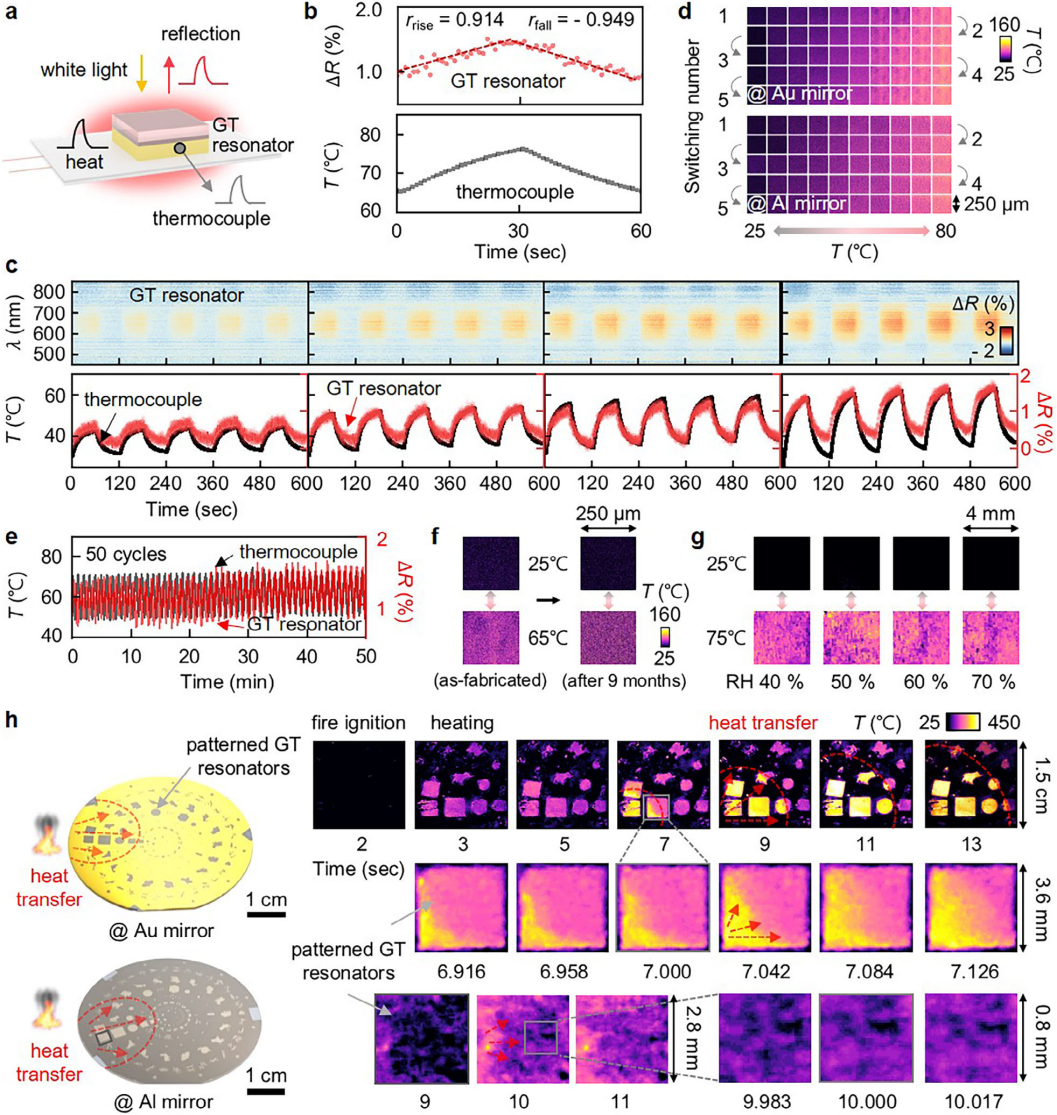
Temperature visualization using GT resonators. a) Experimental setup for temperature monitoring with the GT resonator, together with a thermocouple as a reference. b) Corresponding *ΔR* changes from the GT resonator (top) compared to the temperature measured by the thermocouple (bottom). c) Reversible switching in reflection spectra of the GT resonator during temperature cycling (top: *ΔR* spectra in 2D, bottom: *ΔR* intensity at a wavelength of 650 nm). d) Associated visual representation of the reversible changes in reflection images. e) Reliability during 50 heating and cooling cycles, f) long‐term stability, g) color dynamics under different ambient humidity of the GT resonators. h) Heat transfer visualization across the wafer, demonstrated using pre‐patterned GT resonators on Au mirror (top) and Al mirror (bottom).

The observed volatile characteristics of Te enable an autonomous reverse transition back to the solid phase, allowing for fast and reversible switching dynamics (**Figure** **3**). Given this feature and the critical importance of early detection of battery temperature changes, we evaluate the switching performance of the GT resonator by measuring its spectral changes during both heating and cooling, in 10 °C increments, while simultaneously monitoring the temperature with a commercial thermometer (Figure 3a,b). In both heating and cooling cycles, the GT resonator successfully tracks temperature variations without noticeable delay, achieving a switching speed limited only by the measurement intervals, i.e., 1 second (Figure 3b). A linear fit of the *∆R* values against temperature yields Pearson correlation coefficients exceeding 0.9 for both heating and cooling, indicating a strong linear correlation between optical response and measured temperature.^[^ ^60^ ^]^ Notably, this response speed could be further enhanced, as Te has previously demonstrated nanosecond‐scale switching capabilities.^[^ ^44^ ^]^ The GT resonators also demonstrate highly repeatable and reversible thermochromic performance across multiple cycles and temperature ranges, including room temperature to 40, 50, 55, and 60 °C, with no observable delay (Figure 3c). These dynamics are visibly manifested through reflection image sequences showing consistent color transitions (Figure 3d) and are sustained over at least 50 consecutive heating and cooling cycles (Figure 3e). Furthermore, the thermochromic response remains stable even after nine months of storage (Figure 3f), highlighting long‐term reliability. The resonators also maintain consistent performance under varying relative humidity levels (40%, 50%, 60%, and 70%), confirming their environmental robustness (Figure 3g).

To simulate a practical thermal runaway scenario, we expose the edge of a GT resonator‐coated substrate to an open flame. The resonators successfully visualize heat propagation across a 2‐inch wafer through distinct and progressive color changes (Figure 3h and Supplementary Videos S1,S2, Supporting Information), demonstrating their capability for real‐time, spatially resolved thermal warning. In addition to qualitative visualization, the GT resonators enable quantitative thermal monitoring with a rapid response time of 17 ms and a spatial resolution of 35 µm. This exceptional switching fidelity, combined with scalability and long‐term stability, positions the GT resonator as a strong candidate for integration into next‐generation battery systems for real‐time, distributed temperature monitoring.

### 2.4 Battery Temperature Visualization Using GT Resonators

**Figure 4:**
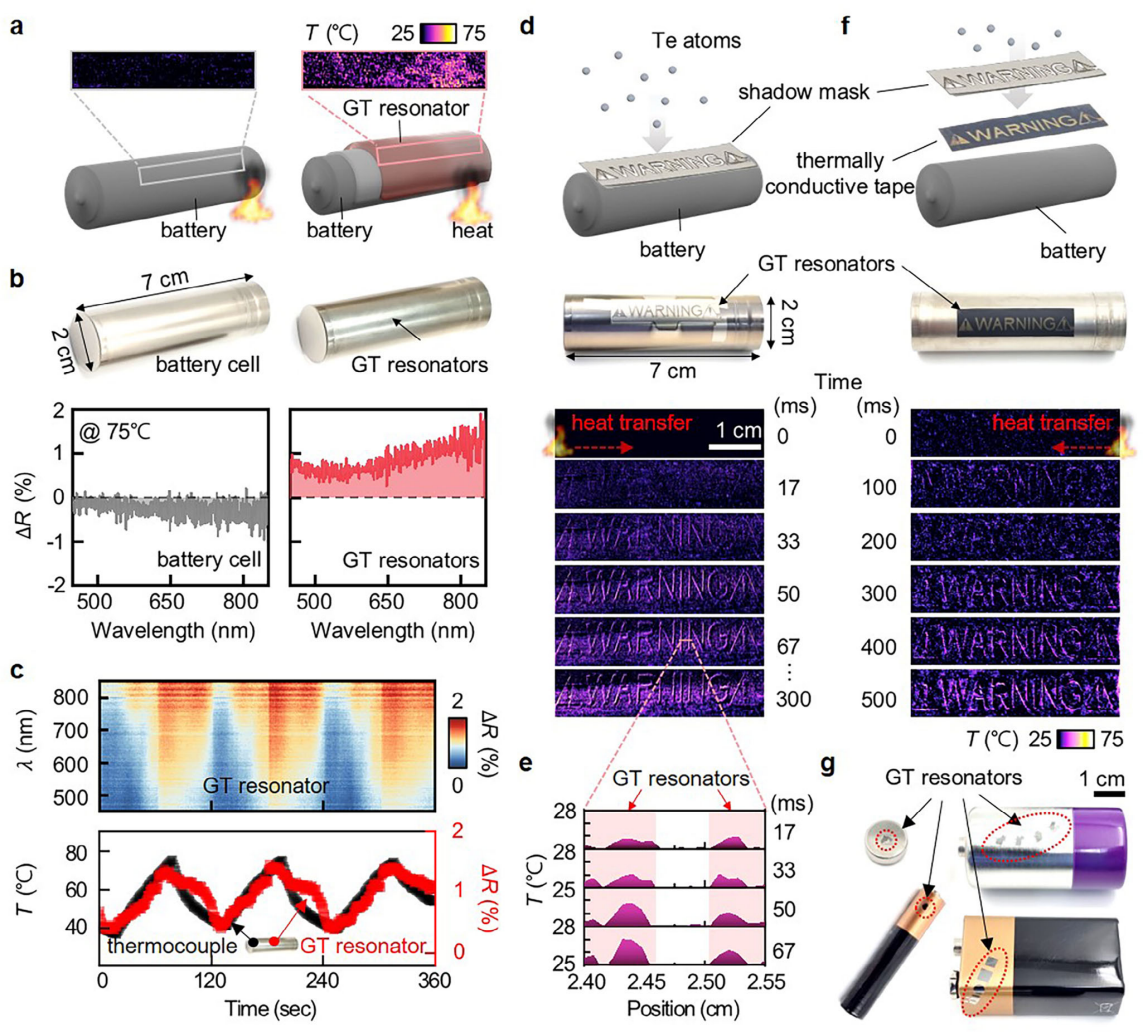
Battery temperature monitoring with GT resonators. a) A camera‐based visualization of battery thermal runaway warning using the GT resonator on the battery surface (right), compared with one without GT resonator (left). b) Reflection images (top) and spectra (bottom) of bare battery (gray) and GT resonator fabricated along the whole battery surface (red). c) The reversible operation of the GT resonator on the battery (top: *ΔR* spectra in 2D, bottom: *ΔR* intensity at a wavelength of 650 nm). d) Heat transfer visualization across the battery surface using “warning” patterned GT resonators deposited directly on the battery surface and e) associated spatiotemporal temperature increase. f) Heat propagation visualization using a thermally conductive tape patterned with the GT resonators attached on the battery surface. g) Various types of batteries with patterned GT resonators.

Accurately monitoring and responding to temperature fluctuations in real time is critical for preventing thermal runaway, making the thermochromic GT resonator a promising tool for enhancing the safety and performance of modern energy storage devices. To demonstrate this capability, we integrate the GT resonator onto the Al shell of a commercially used battery (**Figure** **4a**; Figure S13, Supporting Information). This integration enables the monitoring of heat distribution, a feature unavailable in bare batteries. Despite the rough and curved surface of the Al shell (Figure S14a,b, Supporting Information), the straightforward physical deposition method enables uniform coverage of the Te and SiO_2_ layers across half the battery surface, producing vivid reflective colors (Figure 4b). In contrast, the bare battery exhibits no reflection changes when its surface temperature increases to 80 °C. Although the battery's rough surface leads to lower reflectance compared to one fabricated on the planar silicon wafer (i.e., flat and uniform Al mirror, Figure S14c, Supporting Information), the thermochromic GT resonator on the battery exhibits the effective thermochroic behavior, with a 0.7% Δ*R* change as the temperature varies within the warning region, maintaining strong reversible fidelity (Figure 4c). Given that the temperature difference between a battery's internal and external surfaces is typically < 4–5 °C (Figure S15, Supporting Information),^[^ ^63^ ^]^ integrating the GT resonator onto the battery surface offers an effective solution for early thermal runaway detection.

To provide users with fast and intuitive recognition of temperature increases, we fabricate a “warning” patterned GT resonator directly onto the battery surface using a shadow mask (Figure 4d). This design enables vivid visualization of temperature changes with a temporal resolution of 17 ms. Moreover, unlike thermocouple‐based sensing that measures localized temperatures only (Figure S16, Supporting Information), the thermochromic GT resonator allows clear visualization of heat distribution across the entire battery surface via distinct color variations (Figure 4e; Supplementary Video S3, Supporting Information). This confirms rapid heat transfer within seconds, a capability that is difficult to achieve with point‐based fiber‐optic sensing,^[^ ^5^ ^]^ thermocouples,^[^ ^10^ ^]^ or slow thermo‐responsive phase‐change materials.^[^ ^19^, ^20^ ^]^ To further enhance the practicality of GT resonators, we fabricate patterned versions on commercial thermally conductive tapes (≈1.4 W m^−1^·K^−1^, Figure 4f; Supplementary Video S4, Supporting Information) and polyethylene terephthalate (PET) label stickers (≈0.15 W m^−1^·K^−1^, Figure S17, Supporting Information),^[^ ^61^ ^]^ which are then applied to battery surfaces. Although the PET label slightly hinders heat propagation due to its low thermal conductivity,^[^ ^62^ ^]^ resulting in spatial visualization within tens of seconds (Supplementary Video S5, Supporting Information), the resonators on conductive tape enable heat distribution visualization within 1 s. A key advantage of these GT resonator‐coated stickers is their ease of application to commercial batteries of various shapes and sizes (Figure 4g), highlighting their strong potential for scalable deployment in modern electronic and energy storage systems.

**Figure 5:**
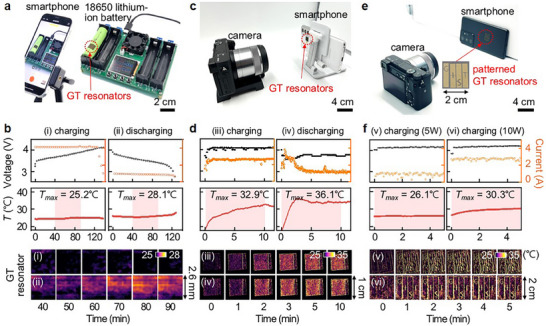
Real‐time temperature monitoring of commercial batteries using GT resonators. a) GT resonator on a rechargeable 18650 lithium‐ion battery. b) Real‐time spatial temperature visualization during (i) charging and (ii) discharging (top panels: voltage and current profiles, temperature measured by a surface‐mounted thermometer (middle panels) and the GT resonator (bottom panels)). c) GT resonator attached to the backside of a commercial smartphone. d) Real‐time temperature monitoring of the smartphone during (iii) charging and (iv) discharging (app operation). e) GT resonator applied to another smartphone. f) Visualization of surface temperature changes during charging with different power inputs: (v) 5 W and (vi) 10 W.

To evaluate the real‐time temperature sensing capabilities of the GT resonators under practical operating conditions, we attach GT resonators fabricated on thermally conductive tapes to commercially available rechargeable systems, including a cylindrical 18650 lithium‐ion battery and a smartphone. The 18650 battery with the GT resonator is connected to a commercial battery cycler to perform controlled charging and discharging tests (**Figure** **5a** and Supplementary Video S6, Supporting Information). The GT resonators successfully detect surface temperature variations resulting from electrochemical reactions within the battery, capturing subtle temperature increases of less than 5 °C during both (i) charging and (ii) discharging processes over a 2‐hour operation (Figure 5b). These results align well with the temperature readings from a surface‐mounted thermometer, clearly demonstrating that the GT resonators can effectively visualize temperature changes under normal battery operation.

We further validate the functionality of the GT resonators by applying them to the backside of a commercial smartphone (Figure 5c; Supplementary Video S7, Supporting Information). During charging at a rate of 10 W, the surface temperature increases to a maximum of 32.9 °C, and corresponding reflectance changes are tracked in real‐time. In addition, when the smartphone is discharged by running a mobile application, the temperature rises more rapidly, clearly visualizing the faster thermal response and enabling intuitive observation of the spatial temperature distribution changes (Figure 5d). During charging with commercial adapters rated at (v) 5 W and (vi) 10 W, the resonators clearly visualized differences in the rate of surface temperature rise, reaching 26.1 and 30.3 °C, respectively, demonstrating sensitivity to varying power input (Figure 5e,f; Figure S18 and Supplementary Video S8, Supporting Information). These findings confirm that while the battery casing may moderately attenuate heat transfer from the internal core to the surface, the GT resonators provide a practical, real‐time, and non‐invasive solution for monitoring temperature dynamics in commercial battery‐powered electronics.

## 3 Conclusion

We successfully demonstrate a tellurium (Te)‐based thermochromic Gires‐Tournois (GT) resonator for rapid and precise temperature monitoring in batteries, particularly within the critical pre‐runaway region below 80 °C, where early detection is essential for risk mitigation. The simplicity of the GT resonator design, combined with a scalable and cost‐effective fabrication process, ensures its practical applicability across a wide range of commercial battery systems. Elemental Te serves as a key thermo‐responsive material, effectively modulating the refractive index in the visible spectrum in response to thermal input. While future work could focus on enhancing color performance through multi‐layered GT resonator designs, such as distributed Bragg reflectors,^[^ ^64^ ^]^ or by exploring alternative materials for passivation and mirror reflection, the current GT resonator already demonstrates the ability to monitor battery temperatures with sub‐second accuracy. This response time is comparable to commercial thermocouples, with the key additional advantage of providing an intuitive, untethered visualization of temperature distribution across the battery surface. Moreover, the material cost per GT resonator, even when applied to one‐half of widely used cylindrical battery types, remains economically viable, supporting its potential for large‐scale deployment in industrial settings (Table S2, Supporting Information). To ensure long‐term stability and robust operation across various use environments, further investigation is warranted into the GT resonator's performance under diverse thermal and mechanical conditions,^[^ ^9^, ^65^ ^]^ with particular attention to safety considerations. While elemental Te is known to be mildly toxic, it is non‐flammable and has been evaluated as a safe material for integration into battery systems.^[^ ^46^, ^66^, ^67^, ^68^ ^]^ Finally, the potential compatibility of this technology with fiber‐optic remote sensing schemes offers exciting possibilities for simultaneous monitoring of an array of batteries, paving the way for advanced thermal management in next‐generation batteries.^[^ ^5^, ^69^ ^]^ Such innovations hold promise for a variety of safety‐critical applications, including heat pipes, wearable devices, robotics, aerospace, electric vehicles, smart building, and automotive systems.^[^ ^12^, ^70^, ^71^ ^]^

## 4 Experimental Section

### Numerical Simulation

Optical properties of GT resonators were calculated using a commercial Finite‐Difference Time‐Domain (FDTD) simulation program (Ansys Lumerical solution 2024 R2 version). The incident light was illuminated as a plane wave propagating toward the GT resonator with a normal incidence for reflectance. The reflectance spectra were obtained ranging from 450 to 850 nm with ≈3 nm intervals. The refractive indices of Te used for simulation were obtained experimentally by spectrometric ellipsometer (Elli‐SEU, Ellipso Technology), covering a spectral range from 450 to 850 nm, with a step size of 1 nm. The incident beam of the ellipsometer was positioned at a 70° to the surface of the resonator. The optical properties of SiO_2_, Au, and Al were extracted from the literature.^[^ ^41^ ^]^ The Te thin film thickness varied, ranging from 1 to 50 nm with 1 nm intervals, to find the maximum reflectance difference.

### Fabrication of the GT Resonator and Integration with Battery

A 150 nm‐thick Au film was deposited on a 2‐inch silicon wafer using electron beam evaporation (KVE‐4000, Korea Vacuum Tech.) at a base pressure of 5 × 10^−6^ Torr and a deposition rate of 0.1 nm s^−1^. Prior to this, a 15 nm‐thick Ti adhesion layer was deposited using the same method. Subsequently, a 10 nm‐thick Te film was deposited onto the Au layer via thermal evaporation at a rate of 0.1 nm s^−1^, with the substrate maintained at −35 °C and azimuthally rotated at 300 rpm during deposition to ensure uniformity. A 50 nm‐thick SiO_2_ layer is then deposited on top of the Te film via electron beam evaporation under substrate cooling conditions, also at a growth rate of 0.1 nm s^−1^. For wafer‐scale structural patterning (e.g., Figure 3h), a 2‐inch aluminum shadow mask fabricated through electroplating (INEXJK.CO.) was positioned in front of the substrate during GT resonator deposition. For battery integration, “warning”‐patterned GT resonators were fabricated using a customized shadow mask produced with a microscale laser cutter (LPKF ProtoLaser U4, LPKF Laser & Electronics). These resonators were deposited either directly onto the surface of an empty commercial battery frame (Sangsin EDP Corporation) or onto commercial polyethylene terephthalate (PET) label stickers (LCC 3130, Formtec) and thermally conductive tapes (9876B‐08, 3m). When fabricated on labels or tapes, the GT resonators were subsequently transferred and adhered to the battery surface for temperature monitoring. The fabricated resonators onto thermally conductive tapes were also applied to a commercial 18650 lithium‐ion battery with energy capacity of 3000 mAh (LYS18650) and a smartphone (Galaxy S21 Ultra, SAMSUNG) to realize their real‐time temperature monitoring under actual battery operation. The 18650 battery was connected to a commercial battery cycler (tester 18650) (operation voltage: DC 5 V, maximum current: 1A) to conduct both charging and discharging processes. The structures of GT resonators were imaged using scanning electron microscopy (SEM, Verios 5 UC, Thermo Fisher Scientific) under an accelerating voltage of 10 kV. Also, the cross‐sectional views of SEM images were used to measure the thickness of the GT resonator. The chemical bonding states of Te and SiO_2_ were analyzed using X‐ray photoelectron spectroscopy (XPS, NEXSA, Thermo Fisher Scientific), X‐ray diffractometer (XRD, SmartLab, Rigaku), TEM (JEM‐ARM300F2, JEOL), and X‐ray energy dispersive spectroscopy (EDS, JED‐2300T, JEOL).

### Thermochromic Characterization

The GT resonators were placed on a commercial microheater (MC‐2550, Sakaguchi electronic heaters) for *in*‐*situ* heating analysis. The complex refractive index of Te thin film under heating was measured using a spectrometric ellipsometer coupled with the microheater setup. Optical images and reflection spectra of the GT resonators during heating were captured using a CMOS camera (STC‐MCS500U3V, Sentech) and a spectrometer (Ocean Optics QEpro) via a customized microscope (Olympus BX53) equipped with 10× (0.3 NA) and 20× (0.45 NA) objectives (Olympus LMPLFLN‐BD). The spectrometer records reflection spectra of the GT resonators at intervals shorter than 1 second. A halogen lamp (PHILIPS 7724, 100 mW) was used as the white light illumination source. Optical images are processed using ImageJ software by isolating the red color channel, which corresponds to the wavelength range of maximum thermochromic response. To examine the thermochromic behavior of the GT resonators at sub‐room temperatures, an aluminum plate connected to a refrigerated circulating chiller (RW3‐0525, ZEIO TECH) was employed. For testing under different humidity conditions, a commercial humidifier was integrated into a sealed, custom‐designed acrylic chamber, allowing controlled adjustment of relative humidity around the GT resonators. Raman spectroscopy (LabRAM HR Evolution, Horiba) was used to confirm the partial melting behavior of Te under heating, with a 532 nm excitation laser. Throughout all *in*‐*situ* heating experiments, sample temperature was monitored using thermocouples (5SC‐TT‐K‐40‐36, OMEGA) connected to a commercial thermometer (OM‐CP‐QUADTCTEMP‐A2, OMEGA), capable of recording temperature at sub‐second intervals.

## Conflict of Interest

The authors filed one patent related to the principle and fabrication method.

## Author Contributions

H.M.K. and H.‐H.J. designed and developed the experiments, with theoretical support from J.H.K and Y.M.S; H.M.K. and G.K. performed the numerical simulation; H.M.K., J.K., G.K., and J.‐H.H. performed the sample fabrication; J.K. and J.L. installed optical setups and thermometer systems; H.M.K. and J.L. conducted optical measurement, analyzed the signals and data; H.M.K., Y.M.S. and H.‐H.J. wrote the paper and all authors contributed editing.

## Supporting information



Supporting Information

Supplemental Video 1

Supplemental Video 2

Supplemental Video 3

Supplemental Video 4

Supplemental Video 5

Supplemental Video 6

Supplemental Video 7

Supplemental Video 8

## Data Availability

The data that support the findings of this study are available from the corresponding author upon reasonable request.
