# Supplementary material for: Thermochromic Gires‐Tournois Resonators with Tellurium for Battery Thermal Runaway Warning
Source: Adv Mater. 2025 Jul 23;37(40):2511261. doi: 10.1002/adma.202511261 (PMC12510277; doi:10.1002/adma.202511261)
Supplement: Supplementary file 1 — Supporting Information [file ADMA-37-2511261-s005.docx]

Copyright WILEY-VCH Verlag GmbH & Co. KGaA, 69469 Weinheim, Germany, 2024.

Supporting Information

Thermochromic Gires-Tournois resonators with tellurium for battery thermal runaway warning

Hyun Min Kim,^1^ JuHyeong Lee,^1^ Juhwan Kim,^1^ Gyurin Kim,^1^ Jang-Hwan Han,^1^ Joo Hwan Ko,^1^ Young Min Song,^1,2,3,4*^ Hyeon-Ho Jeong^1,2,*^

^1^ Department of Electrical Engineering and Computer Science, Gwangju Institute of Science and Technology, Gwangju, 61005, Republic of Korea

^2^ Department of Semiconductor Engineering, Gwangju Institute of Science and Technology, Gwangju, 61005, Republic of Korea

^3^ Artificial Intelligence (AI) Graduate School, Gwangju Institute of Science and Technology, Gwangju, 61005, Republic of Korea

^4^ School of Electrical Engineering, Korea Advanced Institute of Science and Technology (KAIST), Daejeon, 34141, Republic of Korea

*Corresponding authors: ymsong@kaist.ac.kr, jeong323@gist.ac.kr

**Keywords**: thermochromic materials, tellurium, Gires-Tournois resonator, battery thermal runaway warning

**Supplementary Video 1.** Spatial heat transfer visualization across the wafer with the speed of 1 frame per second, realized using pre-patterned GT resonators on Au mirror.

**Supplementary Video 2.** Heat propagation visualization on the pre-patterned GT resonator with the speed of 24 frames per second (temporal resolution of 42 ms).

**Supplementary Video 3.** Visualization of temperature distribution across the battery surface with the speed of 60 frames per second (temporal resolution of 17 ms) when using “warning” patterned GT resonators deposited directly on the battery surface.

**Supplementary Video 4.** Spatial heat transfer visualization across the battery surface when using a thermally conductive tape patterned with the GT resonators attached on the battery surface.

**Supplementary Video 5.** Spatial heat transfer visualization across the battery surface when using a label sticker patterned with the GT resonators attached on the battery surface.

**Supplementary Video 6.** *In-situ* visualization of temperature distribution across the commercial 18650 battery surface during the charging and discharging.

**Supplementary Video 7.**  Spatial heat transfer visualization across the smartphone during the charging and discharging.

**Supplementary Video 8.** Spatial heat transfer visualization across the smartphone under different charging powers (5 and 10 W) using patterned GT resonators.


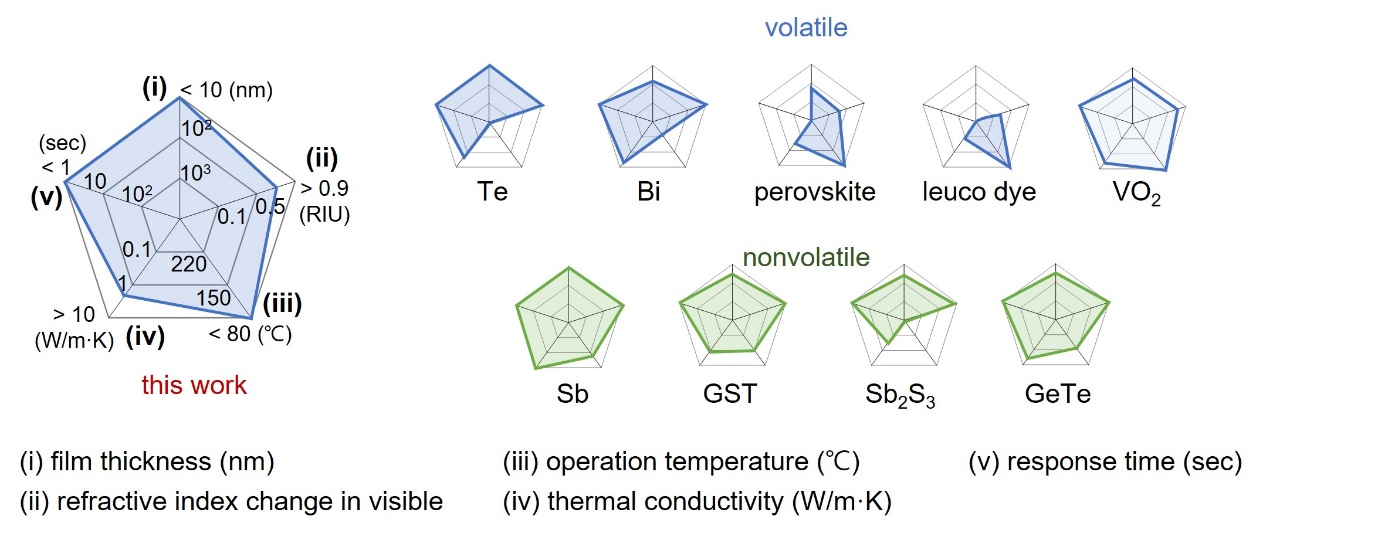


**Figure S1.** Performance comparison between different thermochromic materials.^14–43^


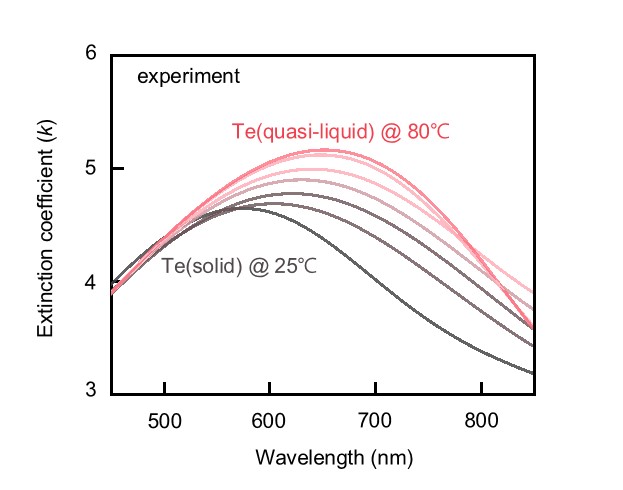


**Figure S2.**  Changes in the extinction coefficient of Te during the phase transition between solid (s) and quasi-liquid (ql) phases.

**
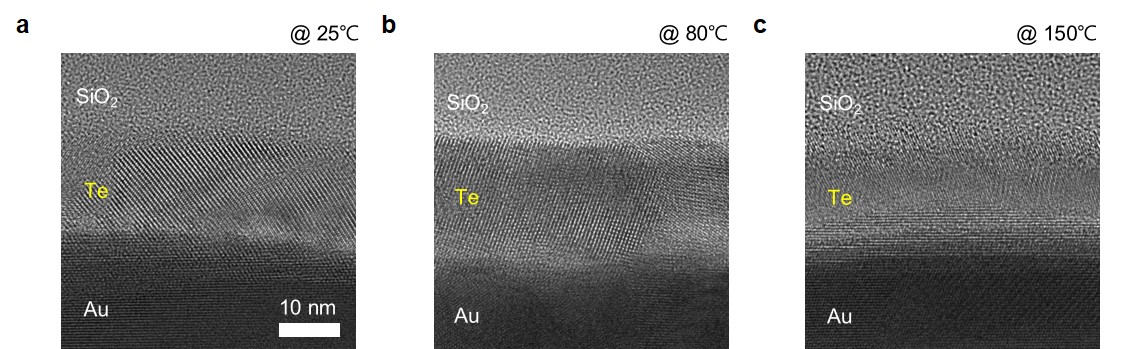
**

**Figure S3.** Cross-sectional TEM images of GT resonators on Au mirror after operating at different heating temperatures, (a) 25^o^C, (b) 80^o^C, and (c) 150^o^C.


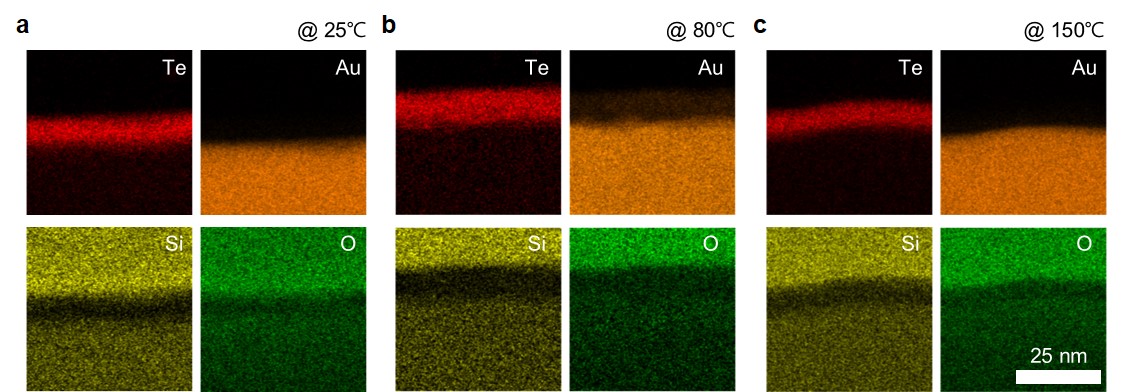


**Figure S4.** EDX elemental false color mapped images of GT resonators on Au mirror after operating at different heating temperatures, (a) 25^o^C, (b) 80^o^C, and (c) 150^o^C.


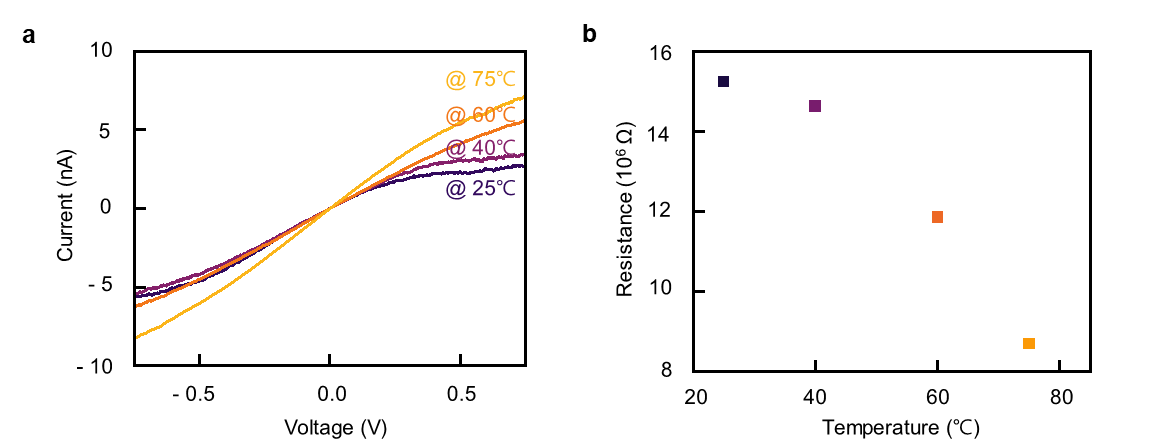


**Figure S5.** Electrical characteristics of Te thin film on electrically insulating quartz substrate when heating. (a) Current-voltage curve and (b) associated variation of electrical resistance with temperature.


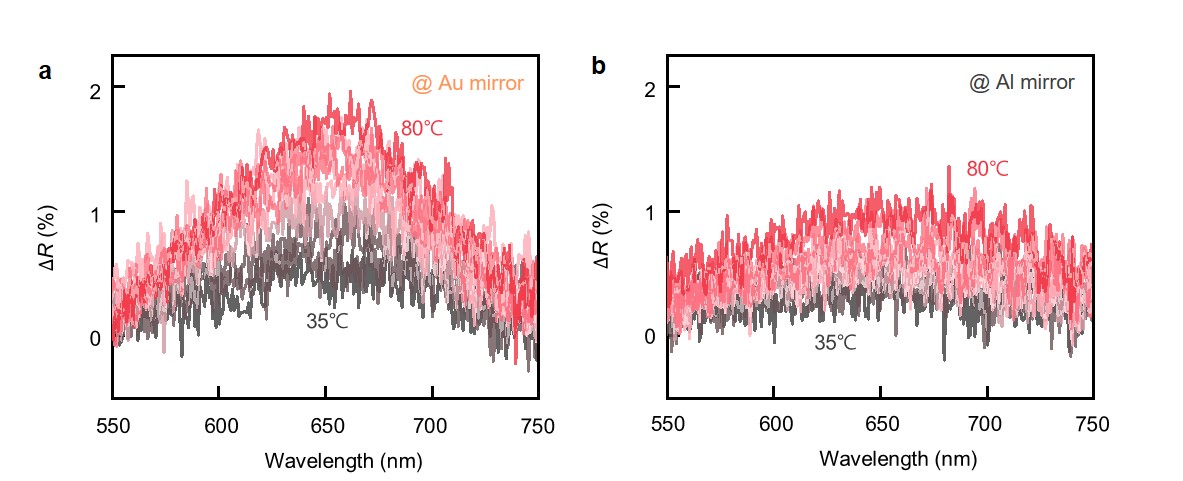


**Figure S6.**  Changes in the reflection spectra of the GT resonators fabricated on (a) Au and (b) Al mirrors when heating them from 35^o^C to 80^o^C.


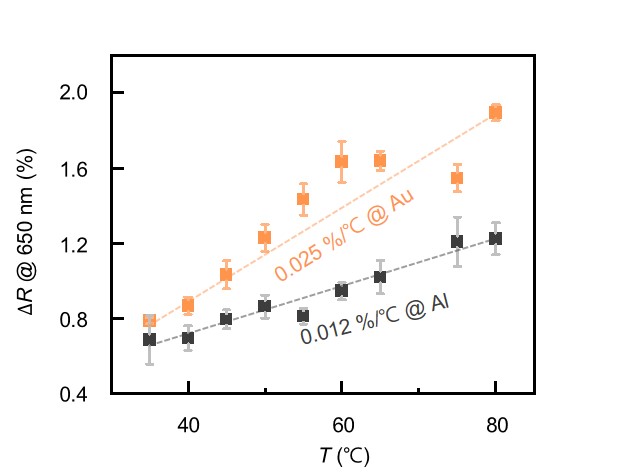


**Figure S7.**  Reflection difference ($\Delta$*R*) values of the GT resonators fabricated on Au (yellow) and Al mirrors (gray) at = 650 nm *vs.* heating temperature.


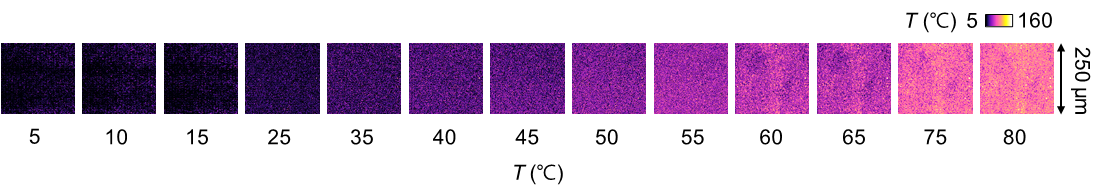


**Figure S8.**  Temperature visualization using the GT resonators when heating them from 5^o^C to 80^o^C.


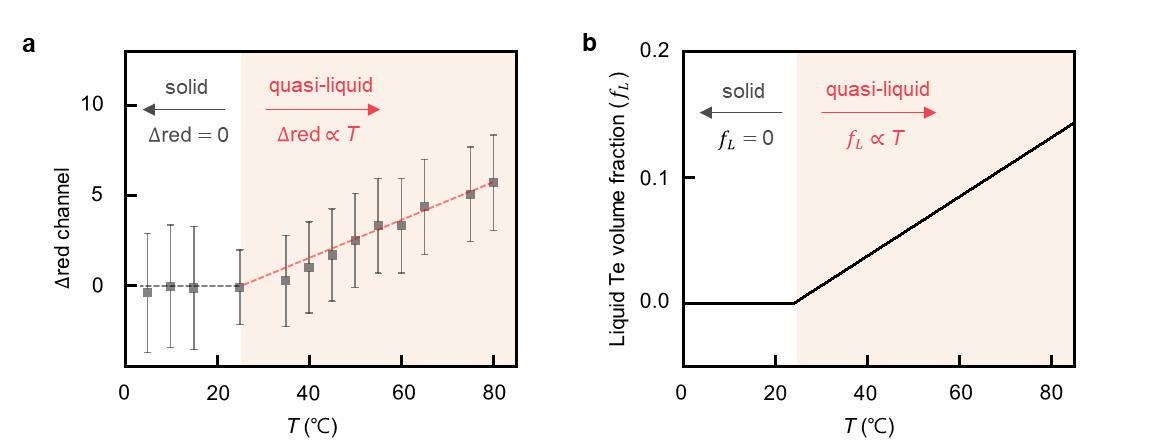


**Figure S9.**  (a) Variation in the red color channel difference ($\Delta$red) of the GT resonator as a function of temperature, and (b) theoretical trend of the liquid Te volume fraction ($f_{L})$ change according to the temperature.


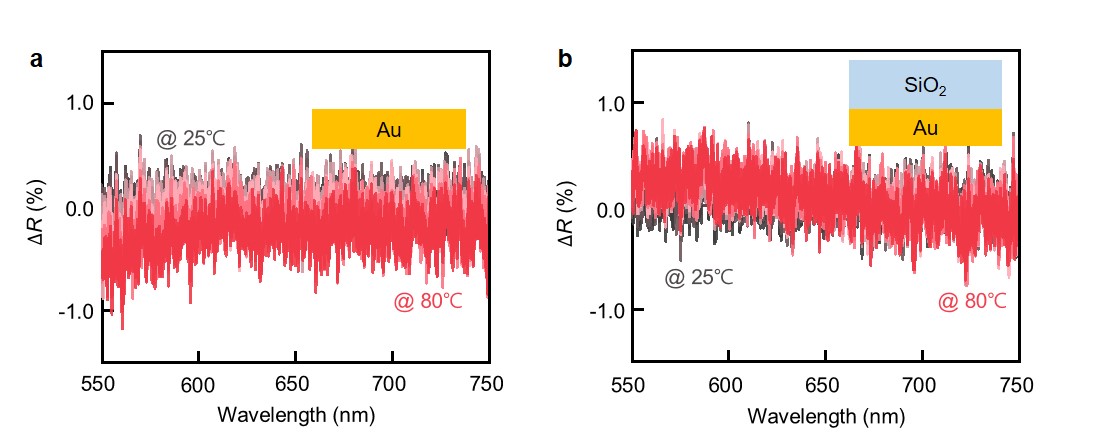


**Figure S10.**  No change in reflection spectra of (a) bare Au mirror and (b) Au mirror coated with a 50 nm SiO_2_ layer when heating them from 25^o^C to 80^o^C.


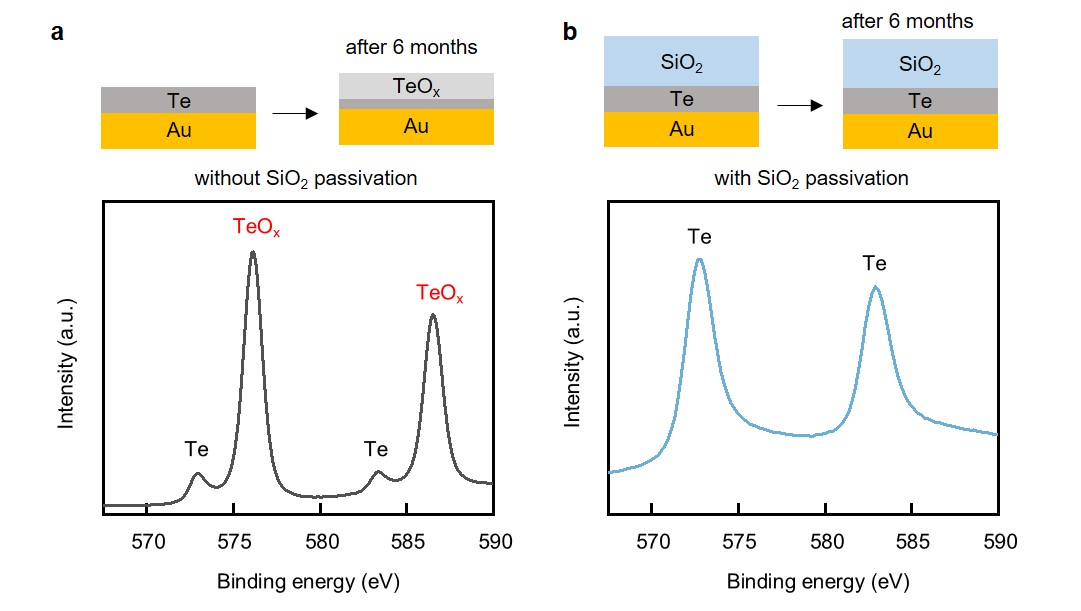


**Figure S11.**  XPS spectra of GT resonators (a) without and (b) with SiO_2_ passivation layer after 6 months in air.


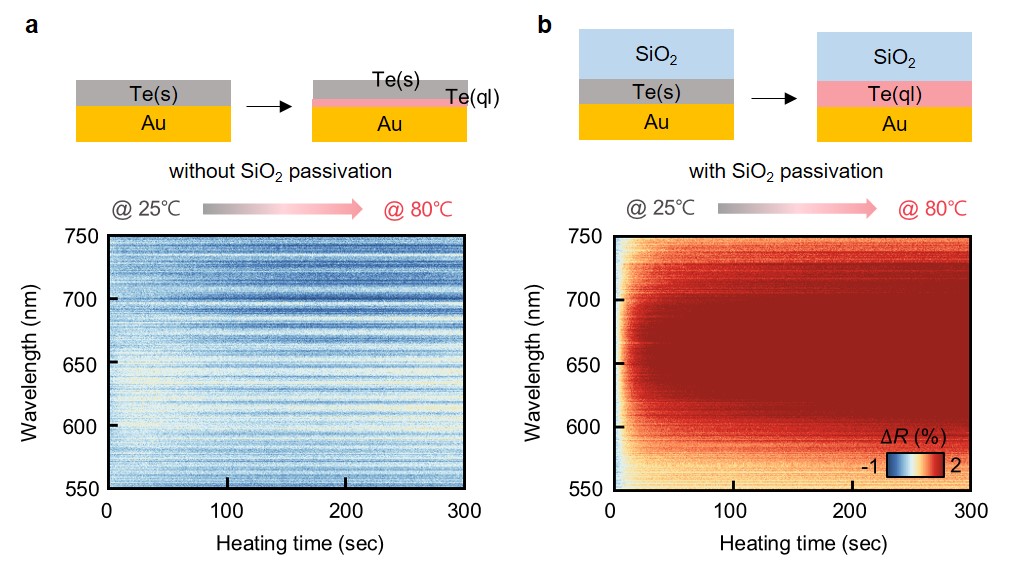


**Figure S12.**  Changes in reflection spectra of GT resonators (a) without and (b) with a 50 nm SiO_2_ passivation layer when heating them from 25^o^C to 80^o^C.


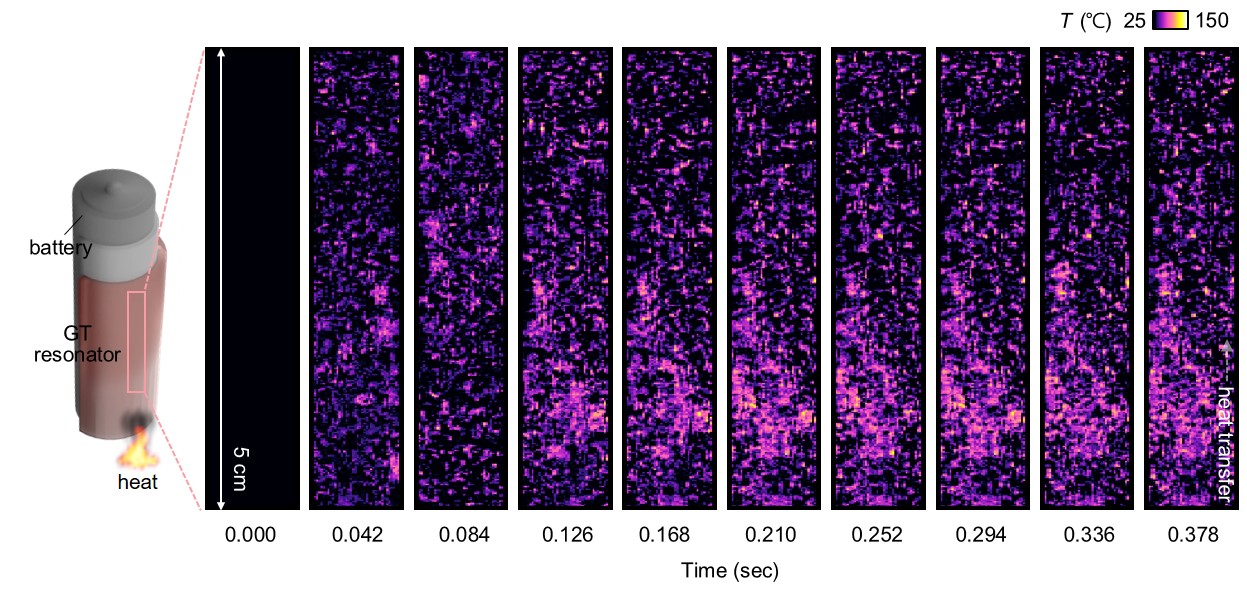


**Figure S13.**  Spatial heat transfer visualization across the battery surface when coated with the GT resonator.


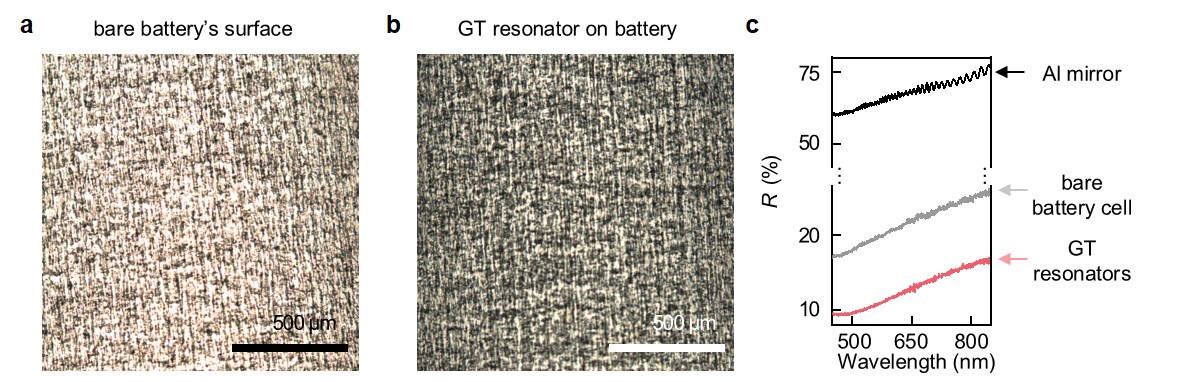


**Figure S14.**  Reflection images of (a) bare battery surface and (b) GT resonator fabricated on the battery surface. (c) Associated reflection spectra, compared with an Al mirror on a planar Si wafer.


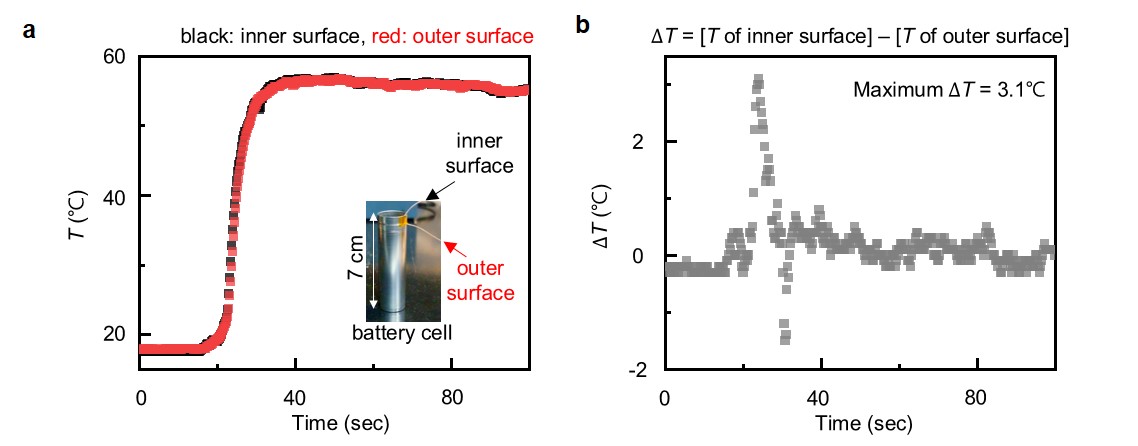


**Figure S15.**  (a) Changes in the temperatures of the inner and outer surfaces of the battery during heating, measured by a thermocouple and (b) associated temperature difference ($\Delta$*T*).


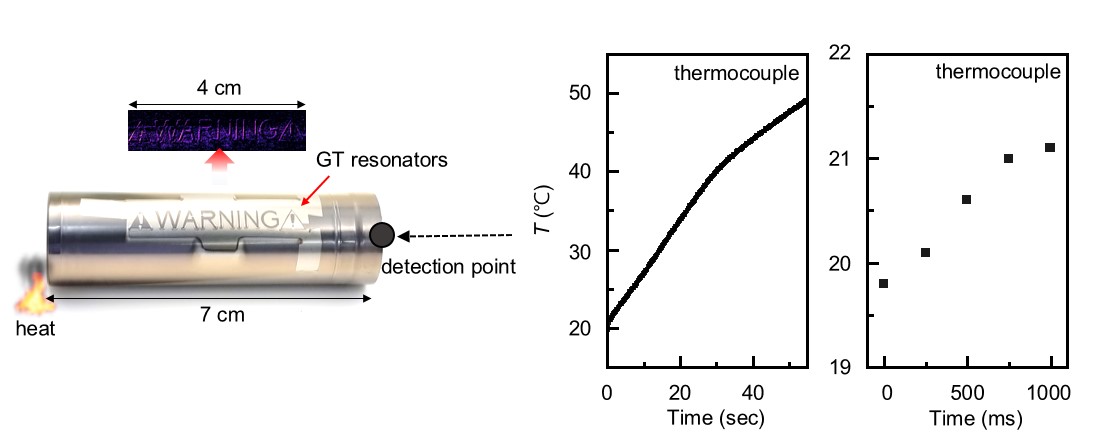


**Figure S16.**  The temperature change in the local area of the battery surface coated with the “warning” patterned GT resonator, measured by a thermocouple, while visualizing the heat transfer in Figs. 4d-e.


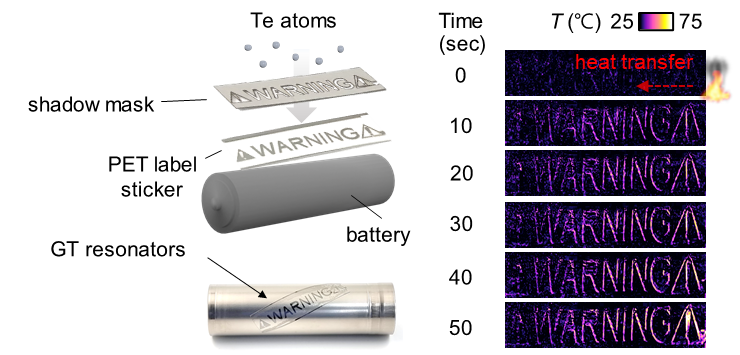


**Figure S17.**  Heat propagation visualization using a commercial polyethylene terephthalate (PET) label sticker patterned with the resonators attached on the battery surface.


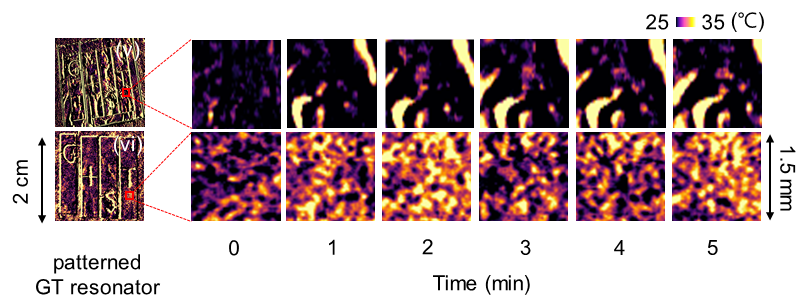


**Figure S18.** Spatial temperature distribution of the GT resonator during charging with (v) 5 W and (vi) 10 W commercial adapters.

**Table S1.** Performance comparison of thermochromic materials.

| No | Material | Working principle | Volatile phase @ RT | Thermal conductivity  (W/m•K) | | Thermal conductance  (MW/m^2^•K) | | Response time | Operation temp.  (℃) | | | max.\|\|  @visible | Ref |
| --- | --- | --- | --- | --- | --- | --- | --- | --- | --- | --- | --- | --- | --- |
| 1 | Liquid crystal | polarization direction tuning | volatile | 0.23 | 0.026  @ 9 m | | 10 ms ~ 21600 s | | | -30 ~ 240 | - | | 14,16,19,  20,39,  S11–S21 |
| 2 | Leuco dye | chemical form transition |  | ~ 0.15 | 1.5  @ 100 nm | | 30 s ~ 86400 s | | | -60 ~ 90 | - | | 14,15,17,18,  S1–S10 |
| 3 | PNIPAM |  |  | ~ 0.51 | 2.6  @ 200 nm | | 1 s  ~ 720 s | | | -10 ~ 60 | - | | S22–S30 |
| 4 | Halide perovskite |  |  | ~ 0.59 | 2.95  @ 200 nm | | 15 s ~ 3600 s | | | 20 ~ 230 | - | | 21,22,  S31–S35 |
| 5 | Sb | crystalline phase change- | non-volatile | 24.3 | 4860  @ 5 nm | | 2 ns ~ 5 s | | | 127 | 1.5 | | 24,32,  S38 |
| 6 | GeSbTe  (GST) |  |  | ~ 1.16 | 38.7  @ 30 nm | | 0.5 ns | | | ~ 150 | 1.1 | | 25,30,31,34,  S39–S41 |
| 7 | GeTe |  |  | ~ 8 | 400  @ 20 nm | | 16 ns | | | 150 ~ 230 | 2 | | 29,31,  S42–S45 |
| 8 | Sb_2_S_3_ |  |  | ~ 0.45 | 22.5  @ 20 nm | | 150 ns ~ 200 ms | | | 312 | 1.1 | | 26,27,30,35,  S46 |
| 9 | VO_2_ |  | volatile | ~ 6.5 | 130  @ 50 nm | | ~ 100 fs | | | 68 | 0.7 | | 28,30,  S47–S49 |
| 10 | Bi | solid - liquid phase change |  | ~ 7.7 | 96.25  @ 80 nm | | ~ 190 fs | | | 230 (nanoparticle)  270  (bulk) | 0.9 | | 23,30,33,  S36,S37 |
| 11 | Te |  |  | 3.38 | 338  @ 10 nm | | 12 ns | | | 450 | 3.5 | | 40-43 |
| This work | Te | Solid - quasi-liquid phase change | volatile | 3.38 | 338  @ 10 nm | | ~ 1 s | | | ~ 80 | 0.71 | | - |

**Table S2.** Cost estimation when fabricating a GT resonator.

**
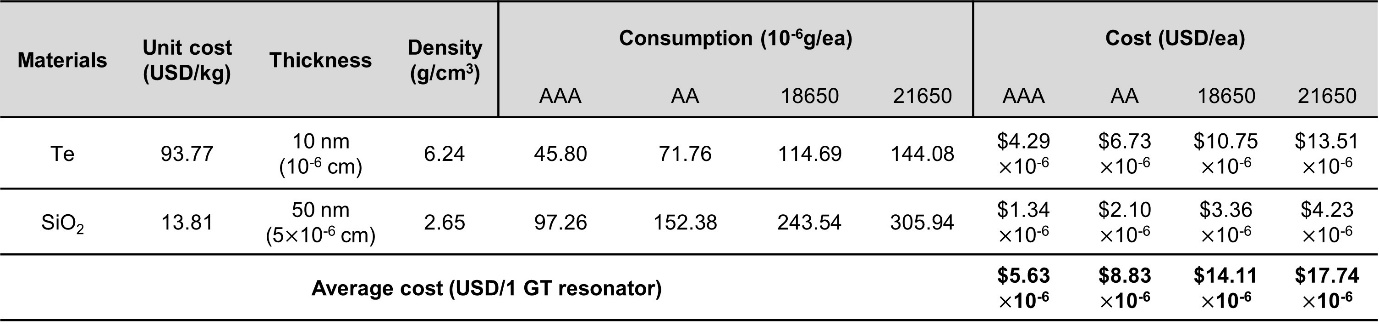
**

**Supplementary References**

S1. Bašnec, K. *et al.* Relation between colour- and phase changes of a leuco dye-based thermochromic composite. *Sci. Rep.* **8**, 5511 (2018).

S2. Panák, O., Držková, M. & Kaplanová, M. Insight into the evaluation of colour changes of leuco dye based thermochromic systems as a function of temperature. *Dye. Pigment.* **120**, 279–287 (2015).

S3. Zhang, W. *et al.* A new approach for the preparation of durable and reversible color changing polyester fabrics using thermochromic leuco dye-loaded silica nanocapsules. *J. Mater. Chem. C* **5**, 8169–8178 (2017).

S4. Panák, O., Držková, M., Svoboda, R. & Klanjšek Gunde, M. Combined colorimetric and thermal analyses of reversible thermochromic composites using crystal violet lactone as a colour former. *J. Therm. Anal. Calorim.* **127**, 633–640 (2017).

S5. Bašnec, K., Hajzeri, M. & Klanjšek Gunde, M. Thermal and colour properties of leuco dye-based thermochromic composite with dodecanol solvent. *J. Therm. Anal. Calorim.* **127**, 55–61 (2017).

S6. Pu, Y. & Fang, J. Preparation and thermochromic behavior of low-temperature thermochromic microcapsule temperature indicators. *Colloids Surfaces A Physicochem. Eng. Asp.* **653**, 129889 (2022).

S7. Spirache, M. A., Marrec, P., Dias Parola, A. J. & Tonicha Laia, C. A. Reversible thermochromic systems based on a new library of flavylium spirolactone leuco dyes. *Dye. Pigment.* **214**, 111208 (2023).

S8. Cartas-Ayala, M. & Karnik, R. Local Temperature Profile Measurement in Microchannels Using Temperature Sensitive Leuco-Dye Microbeads. *Int. J. Micro-Nano Scale Transp.* **2**, 41–56 (2011).

S9. Yu, C. *et al.* All‐Elastomeric, Strain‐Responsive Thermochromic Color Indicators. *Small* **10**, 1266–1271 (2014).

S10. Zhou, W. *et al.* Fabrication and reversible thermotropic characterization of polyurethane/dye microcapsules composite films for thermal management. *Colloids Surfaces A Physicochem. Eng. Asp.* **703**, 135226 (2024).

S11. Strižić Jakovljević, M., Kulčar, R., Friškovec, M., Lozo, B. & Klanjšek Gunde, M. Light fastness of liquid crystal-based thermochromic printing inks. *Dye. Pigment.* **180**, 108482 (2020).

S12. Abdullah, N., Abu Talib, A. R., Jaafar, A. A., Mohd Salleh, M. A. & Chong, W. T. The basics and issues of Thermochromic Liquid Crystal Calibrations. *Exp. Therm. Fluid Sci.* **34**, 1089–1121 (2010).

S13. Zhang, W., Schenning, A. P. H. J., Kragt, A. J. J., Zhou, G. & de Haan, L. T. Reversible Thermochromic Photonic Coatings with a Protective Topcoat. *ACS Appl. Mater. Interfaces* **13**, 3153–3160 (2021).

S14. Abdullah, N., Abu Talib, A. R., Mohd Saiah, H. R., Jaafar, A. A. & Mohd Salleh, M. A. Film thickness effects on calibrations of a narrowband thermochromic liquid crystal. *Exp. Therm. Fluid Sci.* **33**, 561–578 (2009).

S15. Wagner, E. & Stephan, P. Frequency response of a surface thermometer based on unencapsulated thermochromic liquid crystals. *Exp. Therm. Fluid Sci.* **31**, 687–699 (2007).

S16. Irfan, M. *et al.* Thermochromic and highly tunable color emitting bis-tolane based liquid crystal materials for temperature sensing devices. *Dye. Pigment.* **190**, 109272 (2021).

S17. Sentjens, H. *et al.* Programming Thermochromic Liquid Crystal Hetero-Oligomers for Near-Infrared Reflectors: Unequal Incorporation of Similar Reactive Mesogens in Thiol-ene Oligomers. *Macromolecules* **56**, 59–68 (2023).

S18. Kuo, C.-Y., Lin, Y.-T., Huang, T.-T. & Liu, C.-Y. Thermochromic Liquid-Crystalline Elastomers Featuring a Predesigned Hybrid Architecture. *ACS Appl. Polym. Mater.* **6**, 9080–9087 (2024).

S19. Yang, T. *et al.* Thermochromic Cholesteric Liquid Crystal Microcapsules with Cellulose Nanocrystals and a Melamine Resin Hybrid Shell. *ACS Appl. Mater. Interfaces* **14**, 4588–4597 (2022).

S20. Wang, T. *et al.* Polymer Network Film with Double Reflection Bands Prepared Using a Thermochromic Cholesteric Liquid Crystal Mixture. *ACS Appl. Mater. Interfaces* **16**, 18001–18007 (2024).

S21. Meng, W. *et al.* Photothermal Dual Passively Driven Liquid Crystal Smart Window. *ACS Appl. Mater. Interfaces* **14**, 28301–28309 (2022).

S22. Wang, K. *et al.* Thermo-Responsive Poly(*N* -isopropylacrylamide)/Hydroxypropylmethyl Cellulose Hydrogel with High Luminous Transmittance and Solar Modulation for Smart Windows. *ACS Appl. Mater. Interfaces* **15**, 4385–4397 (2023).

S23. Liu, J. *et al.* The thermochromic smart window and thermo-electrochromic device(T-ECD) based on PNIPAm/Ppy composite hydrogel for fast phase transition. *Sol. Energy Mater. Sol. Cells* **263**, 112596 (2023).

S24. Wang, S. *et al.* Strong, transparent, and thermochromic composite hydrogel from wood derived highly mesoporous cellulose network and PNIPAM. *Compos. Part A Appl. Sci. Manuf.* **154**, 106757 (2022).

S25. Jiang, Q. *et al.* Stable and thermochromic organohydrogels for thermostatically controlled display windows. *Chem. Eng. J.* **489**, 151259 (2024).

S26. Guo, R. *et al.* KCA/Na_2_SiO_3_/PNIPAm hydrogel with highly robust and strong solar modulation capability for thermochromic smart window. *Chem. Eng. J.* **486**, 150194 (2024).

S27. Zhang, R. *et al.* Energy-efficient smart window based on a thermochromic microgel with ultrahigh visible transparency and infrared transmittance modulation. *J. Mater. Chem. A* **9**, 17481–17491 (2021).

S28. Feng, Y. *et al.* Phase-Changing Polymer Film for Smart Windows with Highly Adaptive Solar Modulation. *ACS Appl. Mater. Interfaces* **15**, 5836–5844 (2023).

S29. Feng, H. *et al.* Thermally-Responsive Hydrogels Poly(*N* -Isopropylacrylamide) as the Thermal Switch. *J. Phys. Chem. C* **123**, 31003–31010 (2019).

S30. Zhang, Z., Tan, S., Bao, Z., Wu, Y. & Wang, C. Thermal Conductivity of PNIPAm Hydrogels and Heat Management as Smart Windows. *Macromol. Mater. Eng.* **308**, 2200566 (2023).

S31. Rosales, B. A. *et al.* Thermochromic Halide Perovskite Windows with Ideal Transition Temperatures. *Adv. Energy Mater.* **13**, 2203331 (2023).

S32. Lin, J. *et al.* Thermochromic halide perovskite solar cells. *Nat. Mater.* **17**, 261–267 (2018).

S33. Liu, S. *et al.* Near‐Infrared‐Activated Thermochromic Perovskite Smart Windows. *Adv. Sci.* **9**, 2106090 (2022).

S34. Tailor, N. K., Kruszyńska, J., Prochowicz, D., Yadav, P. & Satapathi, S. Thermochromism in Bismuth Halide Perovskites with Cation and Anion Transmutation. *Adv. Opt. Mater.* **12**, 2301583 (2024).

S35. Haeger, T., Heiderhoff, R. & Riedl, T. Thermal properties of metal-halide perovskites. *J. Mater. Chem. C* **8**, 14289–14311 (2020).

S36. Patel, A. & Pandey, S. K. Fabrication of setup for high temperature thermal conductivity measurement. *Rev. Sci. Instrum.* **88**, 015107 (2017).

S37. Jiménez de Castro, M., Cabello, F., Toudert, J., Serna, R. & Haro-Poniatowski, E. Potential of bismuth nanoparticles embedded in a glass matrix for spectral-selective thermo-optical devices. *Appl. Phys. Lett.* **105**, 113102 (2014).

S38. Wei, J. & Gan, F. Thermal lens model of Sb thin film in super-resolution near-field structure. *Appl. Phys. Lett.* **82**, 2607–2609 (2003).

S39. Zhang, W., Mazzarello, R., Wuttig, M. & Ma, E. Designing crystallization in phase-change materials for universal memory and neuro-inspired computing. *Nat. Rev. Mater.* **4**, 150–168 (2019).

S40. Li, Q., Levit, O., Yalon, E. & Sun, B. Temperature-dependent thermal conductivity of Ge_2_Sb_2_Te_5_ polymorphs from 80 to 500 K. *J. Appl. Phys.* **133**, 135105 (2023).

S41. Siegert, K. S. *et al.* Impact of vacancy ordering on thermal transport in crystalline phase-change materials. *Reports Prog. Phys.* **78**, 013001 (2015).

S42. Pries, J. *et al.* Approaching the Glass Transition Temperature of GeTe by Crystallizing Ge_15_Te_85_. *Phys. status solidi – Rapid Res. Lett.* **15**, 2000478 (2021).

S43. Michel, A.-K. U. *et al.* Phase transitions in germanium telluride nanoparticle phase-change materials studied by temperature-resolved x-ray diffraction. *J. Appl. Phys.* **129**, 095102 (2021).

S44. Ghosh, K., Kusiak, A., Noé, P., Cyrille, M.-C. & Battaglia, J.-L. Thermal conductivity of amorphous and crystalline GeTe thin film at high temperature: Experimental and theoretical study. *Phys. Rev. B* **101**, 214305 (2020).

S45. Duan, S. *et al.* Achieving High Thermoelectric Performance by NaSbTe_2_ Alloying in GeTe for Simultaneous Suppression of Ge Vacancies and Band Tailoring. *Adv. Energy Mater.* **12**, 2103385 (2022).

S46. Li, Q. *et al.* Temperature dependent thermal conductivity and transition mechanism in amorphous and crystalline Sb_2_Te_3_ thin films. *Sci. Rep.* **7**, 13747 (2017).

S47. Shao, Z., Cao, X., Luo, H. & Jin, P. Recent progress in the phase-transition mechanism and modulation of vanadium dioxide materials. *NPG Asia Mater.* **10**, 581–605 (2018).

S48. Kizuka, H. *et al.* Temperature dependence of thermal conductivity of VO_2_ thin films across metal–insulator transition. *Jpn. J. Appl. Phys.* **54**, 053201 (2015).

S49. Oh, D.-W., Ko, C., Ramanathan, S. & Cahill, D. G. Thermal conductivity and dynamic heat capacity across the metal-insulator transition in thin film VO_2_. *Appl. Phys. Lett.* **96**, 151906 (2010).
